# Expression of brain-specific angiogenesis inhibitor 1 is inversely correlated with pathological grade, angiogenesis and peritumoral brain edema in human astrocytomas

**DOI:** 10.3892/ol.2013.1250

**Published:** 2013-03-12

**Authors:** WEI WANG, RONG DA, MAODE WANG, TUO WANG, LEI QI, HAITAO JIANG, WEI CHEN, QI LI

**Affiliations:** 1 Department of Neurosurgery, The First Affiliated Hospital of the Medical College of Xi’an Jiaotong University, Xi’an, Shaanxi 710061, P.R. China; 2 Clinical Laboratory, The First Affiliated Hospital of the Medical College of Xi’an Jiaotong University, Xi’an, Shaanxi 710061, P.R. China

**Keywords:** brain-specific angiogenesis inhibitor 1, angiogenesis, peritumoral brain edema, astrocytoma

## Abstract

As the most common intracranial malignant neoplasms, astrocytomas are characterized by high neovascularization and severe peritumoral brain edema (PTBE). Angiogenesis is a prerequisite for the growth of solid tumors, including astrocytoma, and brain-specific angiogenesis inhibitor 1 (BAI1) is a novel angiogenesis inhibitor. In the present study, the expression levels of BAI1, vascular endothelial growth factor (VEGF) and basic fibroblast growth factor (bFGF) were investigated using immunohistochemical methods in 90 human brain astrocytoma specimens of various pathological grades and in 11 normal human brain tissues. Vascular endothelial cells were stained for CD105 and the microvessel density (MVD) was assessed. The volume of astrocytoma and PTBE in each case was evaluated by magnetic resonance imaging (MRI). The results showed that BAI1 was highly expressed in the normal brain tissues, but that the expression decreased with the rising pathological grades of astrocytoma, MVD number and PTBE, indicating that BAI1 expression was inversely correlated with these factors. Furthermore, it was observed that the expression of VEGF and bFGF were inversely correlated with BAI1 expression in the human brain astrocytomas. These results indicate that the BAI1 gene may be used as a marker of decreased tumor progression and tumoral neovascularization, as well as PTBE.

## Introduction

Astrocytoma is the most common intracranial neoplasm and high-grade astrocytoma is characterized by high neovascularization. In high-grade gliomas, endothelial cell proliferation is 40-fold greater than that of normal brain tissue (1). Tumor vessels are characterized by an increased vessel diameter, length, density and permeability. It has been revealed that the development and growth of the tumor neovasculature are tightly regulated by >19 angiogenic factors and 300 antiangiogenic factors (2). Furthermore, the increased levels of angiogenic factors and tumor vessels in astrocytoma lead to severe perilesional edema, while vascular normalization substantially alleviates brain edema in patients with glioblastoma (3,4). Pope *et al* observed that brain edema evaluated by magnetic resonance imaging (MRI) was an independent prognostic factor in patients with malignant gliomas and that patients with gliomas accompanied by severe brain edema often experienced poor clinical outcomes (5).

A novel antiangiogenic gene family, which includes three homologous genes, has been isolated and designated as the brain-specific angiogenesis inhibitor (BAI) family, consisting of BAI1, 2 and 3. BAI1 was isolated from p53 target genes and observed to be expressed specifically in the brain tissue, although this expression was absent in the majority of human glioma cell lines and downregulated in metastatic brain tumors from primary lung adenocarcinoma, indicating that BAI1 may be a tumor suppressor gene for intracranial neoplasms (6,7). BAI1 is a p53 target gene encoding a 1,584-amino acid protein, which is expressed specifically in the brain. Nishimori *et al* observed that wild-type p53 induced the transcription of BAI1, a membrane protein composed of a seven-span transmembrane region and an extracellular domain with five TSP-type 1 repeats which form the functional antiangiogenesis domain of BAI1 (7). Nishimori *et al* demonstrated that BAI1 suppressed the angiogenesis induced by basic fibroblast growth factor (bFGF) in rat corneas using the corneal pocket assay (7). However, the correlation between BAI1 and bFGF and whether the suppression exists in human gliomas remains unknown.

In the present study, the expression of BAI1 was evaluated in normal human brain tissues and astrocytoma specimens of various grades, and then the correlation between BAI1 expression and microvessel density (MVD) labeled with CD105 (endoglin) was analyzed to investigate whether BAI1 may be used as a marker for angiogenesis in astrocytomas. A correlation was identified between BAI1 expression and the expression of two potent angiogenesis factors, vascular endothelial growth factor (VEGF) and bFGF. Immunohistochemistry was used to detect the expression of BAI1, VEGF and bFGF. The experiments were performed using standardized procedures to ensure that the results of the immunohistochemistry could be assessed semiquantitatively (8). The possibility of using BAI1 as a marker for peritumoral brain edema (PTBE) in astrocytomas was also evaluated.

## Materials and methods

### Patients, specimens and tissue preparation

The study cohort consisted of 90 patients with brain astrocytomas, who underwent surgical resection of the tumors at the First Affiliated Hospital of the Medical College of Xi’an Jiaotong University (Xi’an, China) between January 2008 and August 2010. The study was approved by the Ethics Committee of the First Affiliated Hospital of the Medical College of Xi’an Jiaotong University, Xi’an, China. All specimens were obtained from supratentorial surgical resection and divided into four groups according to the World Health Organization (WHO) classification of brain tumors (9) as follows: grade I, 21 cases; grade II, 24 cases; grade III, 27 cases; and grade IV, 18 cases. In total, 11 normal brain specimens, including the cortex and white matter, were obtained at autopsy from patients without any evidence of brain tumors. All patients’ families provided written informed consent prior to enrolment. Within 10 min of surgical resection, the tissues were fixed with freshly prepared 10% formalin at 4°C for 24 h and embedded in paraffin. A pathologist reviewed the astrocytoma specimens to make the pathological diagnoses.

### Immunohistochemistry

The formalin-fixed, paraffin-embedded tissues were serially sectioned to 5-*μ*m thick and collected on poly-L-lysine-coated slides. Staining was performed using immunoperoxidase-staining kits for mouse and rabbit antibodies using goat immunoglobulin.

Following routine deparaffinization and rehydration, the tissue sections were incubated with 3.0% hydrogen peroxide at room temperature (RT) for 10 min to block the endogenous peroxidase. The slides were placed in a glass jar filled with 10 mM sodium citrate buffer (pH 6.0) and boiled for 25 min within a microwave oven to retrieve the antigens. The slides were then incubated with 5% bovine serum albumin (BSA) at RT for 20 min to reduce the non-specific binding. The sections were incubated with the following primary antibodies: rabbit anti-BAI1 affinity purified polyclonal antibody (AB9364, 1:1,000; Chemicon International, Temecula, CA, USA); CD105 (Endoglin) mouse monoclonal antibody (NCL-CD105, 1:100; Novocastra, Newcastle upon Tyne, UK); VEGF (C-1) mouse monoclonal antibody (sc-7269, 1:600; Santa Cruz Biotechnology Inc., Santa Cruz, CA, USA); bFGF (147) rabbit polyclonal antibody (sc-79, 1:800; Santa Cruz Biotechnology Inc., Santa Cruz, CA, USA). Subsequent to incubation with the primary antibodies at 4°C overnight, the sections were rinsed with PBS and incubated with biotinylated goat anti-mouse immunoglobin (for VEGF and CD105), or biotinylated goat anti-rabbit immunoglobulin (for BAI1 and bFGF) at 37°C for 20 min. The sections were then rinsed and incubated with avidin-biotin-horseradish peroxidase complex at 37°C for 20 min. The sections were visualized with 3,3′-diaminobenzidine tetrahydrochloride and counterstained with hematoxylin.

### Evaluation of immunohistochemical staining

Two pathologists, who were blinded to the pathological diagnoses and clinical data, observed the immunohistochemical staining results. A total of 1,000 cells in five random high-powered fields at ×400 magnification were observed per slide and the immunopositive cells for BAI1, VEGF and bFGF were counted. Semiquantitative analysis was performed to determine the expression levels of BAI1, VEGF and bFGF. The labeling index (LI) was defined as the percentage of immunopositive cells (ratio of positively stained cells to the total number of cells per slide, multiplied by 100).

### Identification of MVD by CD105 staining

With the CD105 staining, the MVD of each section was calculated according to Weidner’s criteria (10). Briefly, each section was observed under low-powered fields (LPF) at ×100 magnification to identify the ‘hot spots’. The microvessels of each ‘hot spot’ were then counted in a ×200 magnification field. Single endothelial cells or the clusters of endothelial cells, which were stained dark brown and clearly separated from adjacent astrocytoma cells or connective tissues, were recognized as countable microvessels. A total of five randomly selected fields at ×200 magnification per slide were evaluated and the mean MVD was calculated.

### Assessment of the astrocytoma volume, PTBE and edema index (EI) by MRI

The astrocytoma volume and PTBE of each case were evaluated by MRI (11). The volume of the tumor (V_tumor_) was assessed by gadolinium-diethylenetriamine pentaacetic acid (Gd-DTPA)-enhanced T1-weighted imaging and the volume of the PTBE (V_edema_) was evaluated by T2-weighted or fluid-attenuated inversion-recovery (FLAIR) imaging. In each case, the maximum coronal (a), axial (b) and sagittal (c) radii, which were perpendicular to each other, were measured. The tumor and PTBE volumes were calculated according to the following formula:

V=43πabc

The correlation between the tumor and PTBE volumes in each case was defined as the EI:

$$EI=\frac{V_{\mathrm{tumor}}+V_{\mathrm{edema}}}{V_{\mathrm{tumor}}}$$

### Statistical analysis

The SPSS 13.0 software package was used for all statistical analyses. The data are presented as the mean ± standard error. Differences in the MVD, EI and LI of BAI1, VEGF and bFGF were analyzed among the groups by one-way ANOVA. The correlations between the MVD (or EI) and the LI of BAI1, VEGF and bFGF were analyzed by a Spearman’s rank correlation. P<0.05 was considered to indicate a statistically significant difference.

## Results

### Decreased expression of BAI1 in human astrocytomas of various grades

**Figure 1:**
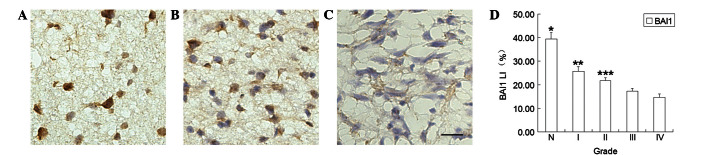
Expression of BAI1 in normal brain tissue and astrocytoma specimens of various grades. The expression of BAI1 was higher in (A) normal brain tissues than (B) low-grade astrocytomas and (C) high-grade astrocytomas. BAI1 expression was higher in (B) low-grade astrocytomas than (C) high-grade astrocytomas. Scale bar = 30 *μ*m. (D) The LI of BAI1 decreased with the pathological grade of the astrocytoma (Spearman’s rank correlation, r=−0.519, P<0.01). ^*^P<0.05 vs. grade I, II, III and IV astrocytomas, ^**^P<0.05 vs. grades III and IV, ^***^P<0.05 vs. grade IV. BAI1, brain-specific angiogenesis inhibitor 1; LI, labeling index; N, normal brain tissue.

Immunohistochemical staining showed that BAI1 was expressed in the cytoplasm of normal human astrocytes and neurons, but only in few human brain astrocytoma cells. The BAI1 expression was high in the normal brain tissues, moderate in the low-grade astrocytomas and low in the high-grade astrocytomas (Fig. 1A–C). There were significant differences between the normal brain tissue and the astrocytomas of the various grades, as well as between grade I and grades III and IV, and grade II and grade IV (P<0.05; Fig. 1D). The BAI1 immunostaining in the astrocytomas decreased with the increasing tumor grades (Fig. 1A–C), and the BAI1 LI was inversely correlated with the pathological grade of the astrocytomas (Spearman’s rank correlation, r=−0.519, P<0.01).

### BAI1 expression is negatively correlated with MVD in human astrocytomas

**Figure 2:**
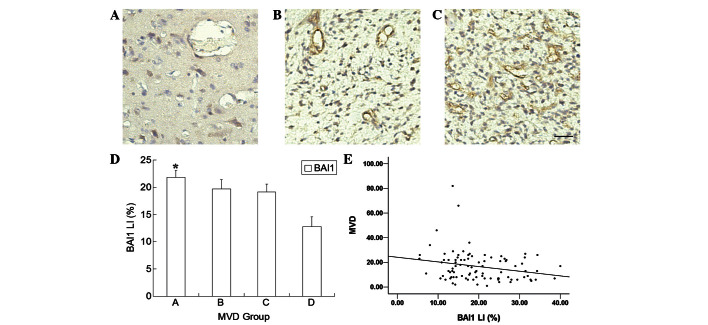
MVD in normal brain tissue and various grades of astrocytomas. MVD was lower in (A) normal brain tissues compared with (B) low-grade and (C) high-grade astrocytomas and higher in (C) high-grade astrocytomas than (B) low-grade astrocytomas. Scale bar = 30 *μ*m. (D) Astrocytoma specimens were divided into four groups according to their MVD value: group A, MVD ≤10; group B, 10< MVD ≤20; group C, 20< MVD ≤30; group D, MVD >30. ^*^P<0.05 vs. group D. (E) The MVD value of the astrocytomas decreased with increasing BAI1 expression (Spearman’s rank correlation, r=−0.222, P<0.05). MVD, microvessel density; BAI1, brain-specific angiogenesis inhibitor 1.

Endothelial cells stained for CD105 were more abundant in the astrocytoma specimens compared with the normal brain tissue. The MVD in the normal brain tissue was significantly lower than that in the astrocytomas (Fig. 2A–C). In the astrocytoma specimens, the MVD decreased as the BAI1 LI increased. To evaluate the correlation between BAI1 and MVD, all astrocytoma specimens were divided into four groups according to their MVD values: group A, MVD ≤10; group B, 10< MVD ≤20; group C, 20< MVD ≤30; and group D, MVD >30. The BAI1 expression in group A was greater than in group D (P<0.05; Fig. 2D). A negative correlation was observed between MVD and BAI1 LI expression (Spearman’s rank correlation, r=−0.222, P<0.05; Fig. 2E).

### BAI1 expression is negatively correlated with the expression of VEGF and bFGF in astrocytomas

**Figure 3:**
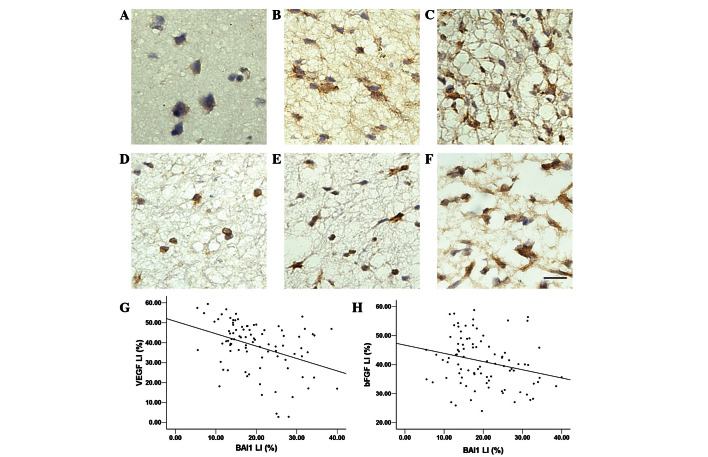
Expression of VEGF was lower in (A) normal brain tissues than in (B) low-grade and (C) high-grade astrocytomas and higher in (C) high-grade astrocytomas than (B) low-grade astrocytomas. Expression of bFGF was lower in (D) normal brain tissues than in (E) low-grade and (F) high-grade astrocytomas and higher in (F) high-grade astrocytomas than (E) low-grade astrocytomas. (G) VEGF expression in astrocytomas decreased with increased BAI1 expression (Spearman’s rank correlation, r=−0.379, P<0.01). (H) bFGF expression in astrocytomas decreased with increased BAI1 expression (Spearman’s rank correlation, r=−0.277, P<0.01). BAI1, brain-specific angiogenesis inhibitor 1; VEGF, vascular endothelial growth factor; bFGF, basic fibroblast growth factor; LI, labeling index.

The BAI1 expression levels were inversely correlated with the tumor neovasculature in the astrocytomas. Furthermore, the correlations between BAI1 expression and two potent angiogenic factors, VEGF and bFGF, were also evaluated. Positive immunostaining for VEGF was observed in the cytoplasm of the endothelial and astrocytoma cells and in small amounts in the astrocytic cells. Normal brain tissue seldomly expressed VEGF, while mild staining was observed in the low-grade astrocytomas and marked expression was observed in the high-grade astrocytomas (Fig. 3A–C). In the astrocytoma tissues, the VEGF LI decreased as the BAI1 LI increased. VEGF expression was negatively correlated with BAI1 expression (Spearman’s rank correlation, r=−0.379, P<0.01; Fig. 3G).

Positive staining for bFGF was observed in the cytoplasm of the astrocytoma, endothelial and astrocytic cells. The expression of bFGF was stronger in the high-grade astrocytomas than the low-grade cytomas and markedly stronger than in the normal brain tissue (Fig. 3D–F). In the astrocytoma specimens, the bFGF LI decreased as the BAI1 LI increased. The expression of bFGF was negatively correlated with BAI1 expression (Spearman’s rank correlation, r=−0.277, P<0.01; Fig. 3H).

### BAI1 expression is negatively correlated with PTBE in astrocytomas

**Fig 4:**
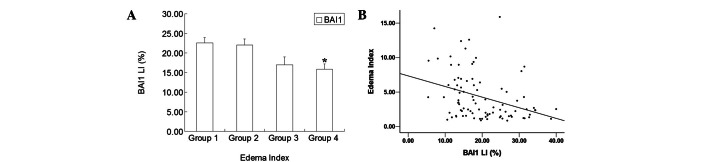
(A) BAI1 expression in astrocytomas was different in patients with various EIs. Astrocytoma specimens were divided into four groups according to their EI: group 1, EI ≤2; group 2, 2< EI ≤4; group 3, 4< EI ≤6; group 4, EI >6. ^*^P<0.05 vs. groups 1 and 2. (B) PTBE in astrocytomas decreased with increased BAI1 expression (Spearman’s rank correlation, r=−0.380, P<0.01). BAI1, brain-specific angiogenesis inhibitor 1; EI, edema index; PTBE, peritumoral brain edema.

Tumoral vessels are characterized by high permeability, which results in severe PTBE. The BAI1 expression was inversely correlated with the tumor neovasculature in the astrocytomas, therefore, its correlation with the PTBE level was investigated. The astrocytoma specimens were divided into four groups according to the EI: group 1, EI ≤2; group 2, 2< EI ≤4; group 3, 4< EI ≤6; and group 4, EI >6. The BAI1 LI of group 4 was significantly lower than groups 1 and 2 (P<0.05; Fig. 4A). The BAI1 expression decreased with the increasing PTBE and was inversely correlated with the EI (Spearman’s rank correlation, r=−0.380, P<0.01; Fig. 4B).

## Discussion

The growth of tumors relies on the formation of their own vascular network (neovascularization), which provides nutrition and oxygen and removes carbon dioxide and other metabolic waste. High levels of vascularization, which contribute to rapid tumor growth and severe PTBE, have been observed in the majority of astrocytomas, particularly in malignant astrocytomas (4). Angiogenesis and antiangiogenesis factors modulate the neovascularization of astrocytomas and also affect PTBE (18). In the present study, the correlations between a novel antiagiogenic factor, BAI1, and vascularization and peritumoral edema were investigated in human brain astrocytomas.

The present results showed that BAI1 was highly expressed in the cytoplasm of astrocytes and neurons in the normal brain tissues, while the expression decreased significantly with the increasing pathological grade in the human astrocytoma specimens. The expression of BAI1 was rare in high-grade astrocytoma specimens, particularly grade IV. These results were consistent with *in vitro* studies, which showed that BAI1 mRNA and protein were absent from the majority of human glioma cell lines (7,12). Izutsu *et al* demonstrated that BAI1 mRNA and protein were downregulated in advanced renal cell carcinoma compared with localized renal cell carcinoma, indicating that BAI1 was crucial for renal cell carcinoma development (13). In addition, BAI1 was observed to be downregulated in pulmonary adenocarcinomas and gastric and colorectal cancers compared with normal tissues (14–17). BAI1 is inversely correlated with the pathological grade of the astrocytoma and may be used as a marker of decreasing malignancy for astrocytomas and other malignant neoplasms.

The MVD was investigated in the human astrocytomas and normal brain tissue and its correlation with BAI1 expression was analyzed. It has been demonstrated that MVD labeled with CD105, which has specific affinity for activated endothelial cells, is a better prognostic factor than either CD31 or CD34 (18–21). Therefore, CD105 was used in the present study as a biomarker for determining the MVD of human astrocytoma specimens and normal human brain tissue. The present study showed that the MVD labeled with CD105 was inversely correlated with BAI1 expression, indicating that BAI1 may be used as a marker of decreased tumoral neovascularization in human astrocytomas.

Other studies have investigated the function of BAI1 in tumoral angiogenesis *in vitro* and *in vivo* and revealed that ectogenic BAI1 suppresses the vascularization of gliomas, pancreatic cancers and renal cell carcinomas (15,22–25). An *in vivo* study showed that an adenoviral vector encoding BAI1-transduced glioblastoma cell xenografts exhibited significant suppression of tumor growth and impairment of tumor angiogenesis (36). However, the antiangiogenic mechanism of BAI1 in human astrocytomas has not been thoroughly investigated. The BAI1 extracellular domain may be separately proteolyzed and cleaved into 120-kDa [Vasculostatin-120 (Vstat120)] and 40-kDa [Vasculostatin-40 (Vstat40)] fragments, which have significant antiangiogenic activities *in vitro* and *in vivo*, depending on the presence of the CD36 receptor on the target endothelial cells (26–28). Moreover, BAI1 has been shown to specifically inhibit endothelial cell migration, which also contributes to the antiangiogenic properties of BAI1 (16).

The balance between angiogenic and antiangiogenic factors is crucial for the clinical prognosis of patients with glioblastomas. VEGF and bFGF are two potent angiogenic factors. Kudo *et al* noted that BAI1 inhibited the angiogenesis of murine renal cell carcinoma by suppressing VEGF expression (23). Nishimori *et al* observed that the neovascularization induced by bFGF was inhibited by BAI1 (7). Nam *et al* demonstrated that patients with glioblastomas with high VEGF and absent BAI1 mRNA expression often experienced poor outcomes (22).

Furthermore, the correlations between the expression levels of BAI1 and two potent angiogenic factors, VEGF and bFGF, were analyzed using the astrocytoma specimens. Despite the degrees of malignancy, the present study showed that the lower the BAI1 expression, the higher the VEGF and bFGF expression levels. Whether the BAI1 expression inhibited the expression of VEGF and bFGF, or vice versa, requires further investigation in the future.

Severe astrocytoma PTBE induces elevated intracranial pressure and leads to poor clinical outcomes. Patients with glioblastoma multiforme without PTBE experienced double the median survival time compared with those with PTBE (5). Higher tumor angiogenesis and vasogenic edema have also been associated with higher morbidity and mortality rates in patients with malignant brain tumors (3).

The present study showed that BAI1 expression was inversely correlated with PTBE in human brain astrocytomas, indicating that BAI1 may be used as a marker of decreased PTBE for patients with this condition. Schoenegger *et al* observed that patients with major edema experienced significantly shorter overall survival times compared with patients with minor edema (29). PTBE was likely to affect morbidity and the invasive potential of malignant gliomas (30). Therefore, patients with higher BAI1 expression may experience minor PTBE and superior overall survival.

BAI1 is a multi-functional protein with crucial roles in cell adhesion and signal transduction in the brain (31). BAI1 has the ability to mediate membrane-cytoskeletal interactions and signal transduction during neuronal growth (32) and also to regulate neurotransmitter release (33). BAI1 is critical for neural development, synapse formation, signal transduction at neuron synapses, cell proliferation and cytokinesis (34). Koh *et al* revealed that BAI1 inhibited neuronal differentiation (35). A clinical study showed that patients with gliomas expressing BAI1 were sensitive to radiotherapy, suggesting that exogenous BAI1 may increase the sensitivity of astrocytomas to radiation (22).

In summary, BAI1 is inversely correlated with pathological grade, angiogenesis and PTBE and may be used as a marker for decreasing malignancy, neovascularization and perilesional brain edema in human astrocytomas. Further research focused on the function of BAI1 should be performed in the future.
